# Reversal of cisplatin triggered neurotoxicity by *Acacia hydaspica* ethyl acetate fraction via regulating brain acetylcholinesterase activity, DNA damage, and pro-inflammatory cytokines in the rodent model

**DOI:** 10.1186/s12906-022-03657-3

**Published:** 2022-07-05

**Authors:** Tayyaba Afsar, Suhail Razak, Ali Almajwal

**Affiliations:** Department of Community Health Sciences, College of Applied Medical Sciences, King Saud University, Riyadh, Saudi Arabia

**Keywords:** Cisplatin, Hydaspica ethyl acetate extract, Acetylcholinesterase activity, Neurodegeneration, Inflammatory markers

## Abstract

**Background:**

Cisplatin (CisPT) is a chemotherapeutic that outcome in adverse effects including neurotoxicity. We examined the efficacy of *hydaspica* ethyl acetate extract (AHE) against CisPT-prompted neurotoxicity.

**Methods:**

Group I: Distilled water; Group II: CisPT (12 mg/kg b.w. i.p) on the 13^th^ day of treatment. Group III: received AHE (400 mg/kg b.w) orally for 16 days. Group IV and V received 200 and 400 mg/kg b.w AHE orally for 16 days while CisPT injection on day 13, respectively. Group VI: received Silymarin (100 mg/kg b.w) orally for 16 days and CP (12 mg/kg b.w., i.p.) on day 13. TNF-α, IL6, brain acetylcholinesterase activity (AChE), oxidative trauma markers, genotoxicity, antioxidant enzymes, and morphological alterations in cerebral hemispheres were inspected.

**Results:**

AHE administration before CisPT considerably reduced both tissue TNF-α and IL 6 expressions compared to CisPT treated group in a dose-dependent manner. AHE treatment (400 mg/kg b.w) significantly ameliorated brain AChE activity. Brain tissue MDA, H_2_O_2,_ and NO content were markedly (*p* < 0.001) elevated after CisPT inoculation while a noticeable (*p* < 0.001) diminution was observed in AHE treatment groups. AHE treatment significantly (*p* < 0.001) improved brain antioxidant defense in a dose-dependent manner. Furthermore, AHE efficiently recused CisPT to induce DNA damage in brain tissue as revealed by ladder assay and DNA fragmentation patterns. Histopathological findings revealed severe neurodegenerations in CisPT treated group, however, AHE treatment noticeably precluded morphological alterations and neuron damages induced by CisPT.

**Conclusion:**

*A. hydaspica* AHE extract may be provided as a prospective adjuvant that precludes CisPT-induced neurotoxicity due to its radical scavenging and antioxidant potential.

## Background

Cis-diamminedicholoroplatinum (Cisplatin/ CisPT) is a platinum-based compound used as a part of standard chemotherapeutics for the treatment of various cancers [1]. Despite substantial anti-cancer efficiency, patients are often overwhelmed with detrimental side effects from the treatment which can lead to dose reduction or even termination of chemotherapy that can risk the patient’s life. Following CisPT cytotoxicity is not specific to only cancerous cells so CisPT reception into healthy cells is higher than in cancer cells, rendering healthy cells more vulnerable to damage [2]. Nephrotoxicity and neurotoxicity are the major downsides that restrict the clinical use of cisplatin at required doses. CisPT penetrated the human brain causing an intensification of CisPT accumulation in the cerebrospinal fluid, where it inhibits neuronal stem cell proliferation and causes oxidative stress in the brain [3]. Moreover, CisPT administration ensues massive cellular damage [4], 8-oxoguanine DNA damage, inflammation, and mitochondrial dysfunction, consequently causing cell-cycle arrest and apoptosis in the nervous system [5]. However, the therapeutic effectiveness of CisPT cannot be over sighted. Hence, the need to discover safe complementary remedies to protect against the neurotoxicity of CisPT is a leading goal of preclinical research. To achieve this goal, understanding the mechanisms of neurological damage triggered by CisPT is essential for the development of therapeutic interventions. As oxidative stress through reduction of plasma antioxidant enzyme levels and inflammation are key hallmarks in the development of CisPT neurotoxicity. Therefore, consuming composites with antioxidant and anti-inflammatory actions may be favorable against medicine-related injuriousness. Several antioxidant agents can reduce cisplatin-induced neurotoxicity [6]. The antioxidant and anti-inflammatory activity of polyphenols has been well studied in vivo and in vitro [7].

Acacia species have been used in medicines, baking components, and carpentry for epochs*. Acacia hydaspica* R. Parker synonyme *Acacia eburnean* (family: Leguminosae) is Pahari Kikar, Kikar; Marmat. The seeds and bark possess a high amount of tannins [8, 9]. *A. hydaspica* exhibited anticancer, antioxidant [10], anti-inflammatory [3], cardioprotective [11], and efficient against CisPT-induced reproductive and hepatic toxicity [12, 13]. GCMS study documented α-Amyrin (5.03%), 1,2-Benzenedicarboxylic acid mono (2-Ethylhexyl) ester (70.65%), Vitamin E (4.56%), Squalene (4%) and 2,6-dimethyl-N-(2-methyl-à-phenyl benzyl) aniline (2.51%) in *A. hydaspica* [1]. Bioassay-guided separation revealed 7-*O*-galloyl catechin, catechin, catechin gallate, and methyl gallate as chief antioxidant and anticancer phytoconstituents from *A. hydaspica* ethyl acetate fraction (AHE) [4, 10, 14]. Genus *Acacia* exposed antioxidant and neuroprotective abilities in animal prototypes [5]. Previous studies reported that green tea polyphenol ( −)-epigallocatechin gallate possesses shielding influence against oxidative stress-induced neurological injuries [15]. Continuing administration of catechin improves spatial learning ability by reducing oxidative stress and improving endogenous antioxidant status [2]. An antioxidant active compound epigallocatechin 3-*O*-gallate; isolated from *Acacia* *mearnsii*, showed neuroprotection against Acrolein-induced oxidative damage by the attenuation of reactive oxygen species [16]. The efficacy of AHE to protect against CisPT‑induced neurotoxicity has not yet been evaluated. This study considered the use of *Acacia hydaspica* against CisPT-induced brain toxicity and oxidative trauma in rats. We hypothesized that polyphenol-rich AHE could preclude CisPT-induced oxidative stress and inflammation due to its antioxidant properties. The effect of AHE on the antioxidant levels, biochemical variations, DNA damage, and histoarchitecture was studied.

## Methods

### Plant sample collection and extraction

*A. hydaspica* (Aerial parts) were collected from the Kirpa Charah area in Islamabad, Pakistan. For the collection of plant material, field studies were conducted following Pakistan legislation. Authors comply with the IUCN Policy Statement on Research Involving Species at Risk of Extinction and the Convention on the Trade in Endangered Species of Wild Fauna and Flora. The plant material was authenticated by Dr. Sumaira Sahreen (Botanist/Curator at Herbarium of Pakistan, Museum of Natural History, Islamabad) and the voucher specimen was deposited (accession No. 0642531) in the Herbarium of Pakistan, Museum of Natural History, Islamabad for future reference. The extract preparation and fractionation were reported in detail in our previous investigations on *A. hydaspica* pharmacological activities [17], and its ethyl acetate extract (AHE) showed maximum bioactivity under in vitro and in vivo investigations and possesses bioactive polyphenols [4, 11, 17]) was selected for the current study.

### Dose preparation

Cisplatin (CisPT) dose (12 mg/kg bw) was preferred to study neurotoxicity potential following published literature [18]. The drug (CisPT injection) was purchased from Sigma-Aldrich (St. Louis, MO, U.S.A.). 200 mg/kg b.w and 400 mg/kg b.w doses of AHe were selected based on our previous lab investigations that confirmed the efficacy of these selected doses [19–21]. Silymarin (100 mg/kg b.w) and AHE (200 and 400 mg/kg b.w) were dissolved in distilled water just before treatment.

### Treatment regimen

Male rats (Sprague Dawley, 200–230 g) were housed in the animal husbandry facility Department of Animal Sciences, Quaid-i-Azam University, Islamabad. The rats were captive in steel cages and fed with the usual pellet diet and tap water. The temperature was maintained at 25 ± 3 ºC under 12 h light/dark cycles. 42 rats were randomly separated into six groups (*n* = 8) and grouped in separate cages. The study procedure was designed following an earlier protocol [22] with slight adjustments. The study is reported under ARRIVE guidelines [23]. The experimental method for the use of animals was authorized (Bch#0256) by the ethical board of Quaid-i-Azam University, Islamabad Pakistan.

- Group I: Given water orally for 16 days, and saline (2 ml/kg, i.p) injection on day 13.
- Group II: One dose of CP (12 mg/kg b.w., i.p.) was inoculated on day 13.^th^
- Group III: Received AHE (400 mg/kg b.w) orally for 16 consecutive days
- Group IV and V: Taken AHE (200 and 400 mg/kg b.w) doses for 16 consecutive days and a single dose of CisPT (12 mg/kg, i.p) on day 13 respectively.
- Group VI: received silymarin (100 mg/kg b.w) orally for 16 consecutive days and CP (12 mg/kg b.w., i.p.) on day 13.

AHE was given for 12 days before CisPT to fortify the bioavailability of test sample components (antioxidant) to ascertain the anti-oxidative immunity of animals before CisPT introduction. The reason for the sacrifice of animals on day 17, after 4 days of CisPT inoculation was to make sure the bioavailability of CisPT stimulated the neurotoxic effect, thereby authenticating the effectiveness of AHE in precluding the neurotoxicity interceded by CisPT.

Body weights of rats were documented at the start and end of the experiment. 24 h after the last dose rats were euthanized by cervical dislocation [24]. This method is declared a humane method of euthanasia by AVMA recommendations for the Euthanasia of Animals: 2013 Edition. Cervical dislocation is a method that may induce rapid loss of consciousness. It does not chemically contaminate tissues [25]. Blood was drawn and serum was extracted, which was later stored at -80 °C for further examination. The cranium was open and the brain was carefully removed. Brain tissue from 4 animals per group was cut into small sections carefully and brain cerebral cortex sections were fixed in 10% neutral buffered formalin. Five-micrometer slices of neutral formalin-fixed and paraffin-embedded tissues were routinely cut by a microtome for histological examination while remaining brain tissues were dried with liquid nitrogen and stored at -80 °C for enzymatic and DNA damage examinations.

### Biochemical testing

#### Tissue homogenization

100 mg of tissue slice was grounded in homogenizer using a mixture of 100 mM KH2PO4 buffer and 1 mM EDTA (pH 7.4). The samples were centrifuged at 12,000 × g for half an hr at 4 °C. The supernatant was separated and used to estimate the level of oxidative stress indicators, antioxidant enzyme status, and acetylcholinesterase activity. Bradford method was used to measure the protein concentration [26].

#### Acetylcholinesterase (AChE) enzyme action

The brain acetylcholinesterase enzyme regulation was measured by the procedure described previously [27]. Briefly, the reaction mixture includes distilled water (135 μL), 100 mM potassium phosphate buffer (pH 7.4, 20 μL), 10 mM DTNB (20 μL), diluted tissue homogenate sample (1:10 v/v, 5 μL), and 8 mM acetylthiocholine (20 μL) as a substrate. The drop in acetylthiocholine iodide activity was recorded for 5 min (30 s intervals) at 412 nm and the data were recorded as μmol/min/mg protein.

#### Measurement of pro-inflammatory cytokines

TNF-α and IL-6 antibody activity was measured both in the brain as well as serum samples by ELISA kit procedure. Absorbance was measured at 450 nm, and antibody expression was measured as pg/mg protein in the brain and as pg/mL when calculated in the serum sample.

#### Assessment of brain enzymatic antioxidants

CAT and POD enzyme activity was monitored in samples following earlier reports [13]. Kakkar et al. protocol was used for the assessment of SOD activity [28]. The quinone reductase levels in tissue samples were calculated as previously designed protocol [29]. Reduced glutathione quantity was estimated as described by Jollow [30]. The technique of Habig et al. [31] was used for the estimation of GST content. Glutathione reductase and Glutathione peroxidase consumption in tissue samples was observed as cited formerly [32, 33]. The utility of γ-glutamyl transpeptidase was checked following Orlowski et al. scheme [34].

#### Assessment of brain oxidative stress indicators

Iqbal et al. procedure [35] were implemented for the calculation of lipid peroxidation. Approximation of the hydrogen peroxide content in the brain tissue was verified by the scheme described earlier [36]. For the execution of nitrite assay, Griess reagent was utilized [37].

### Examination of DNA Damage

#### DNA fragmentation test

Brain tissue (100 mg) was homogenized using TTE mixture (5 mM Tris–HCl + 20 mM EDTA + 0.2% Triton X-100). 100 µl aliquot was poured into the separate tube (B) and centrifuged at 200 × g (4 °C) for 10 min. Over again the supernatant was pipette out in a new tube (S) and centrifuged at 20,000 × g at 4 °C for 10 min to extract the intact chromatin and marked as “T”. Subsequently, 1 ml of 25% Trichloroacetic acid (TCA) was dispensed in all labeled tubes and left overnight at 4 °C. The next day the samples were centrifuged for 10 min at 18,000 × g (4 °C) to recover the precipitated DNA. Then 160 μl 5% TCA was dispensed in each tube and heated for 15 min at 90 °C. DNA content was estimated by adding 320 μl of freshly prepared Diphenylamine (DPA) dye. The changes in optical density were recorded at 600 nm (Smart spec TM Plus, catalog # 170–2525).

The amount of DNA fragmentation was quantified by using the given equation.

% DNA fragmentation = $$\mathrm{Tx}\frac{100}{\left(\mathrm{T}+\mathrm{B}\right)}$$

#### DNA ladder assay

DNA was isolated from tissue samples by standard protocol [25]. Brain tissue (100 mg) was rinsed and homogenized in 1 ml of lysis buffer. 100 μl of proteinase K (10 mg/ml) and 240 μl of SDS (10%) were added to homogenate and stirred slowly and incubated overnight in a water bath at 45 °C. The next day 400 μl of phenol was added to the homogenate mixture and kept in a shaker for 5–10 min and then centrifuged at 3000 rpm (10 °C) for 5 min. The supernatant was separated carefully and diluted with 1.2 ml of phenol + 1.2 ml of a mixture containing chloroform/isoamyl alcohol (24:1, 1.2 ml). The reaction tubes were shaken for 5–10 min and centrifuged at 3000 rpm (10 °C) for 5 min. Subsequently, the supernatant was separated and mixed with 25 μl of sodium acetate (pH 5.2, 3 M) and 5 ml ethanol, and kept on a shaker until DNA was precipitated. DNA was washed with 70% ethanol and uncontaminated DNA was dissolved in TE buffer. The content of DNA was measured at 260 and 280 nm. 5 μg of each sample DNA and 0.5 μg DNA standards were loaded on 1.5% agarose gel. Electrophoresis was run for 45 min at 100 V batteries, and DNA was imaged under a digital gel doc system and photo'd.

#### Histopathology examination

Tissue slices from each group were poured into buffered formalin. After the dehydration procedure, the tissues were fixed in paraffin blocks for microtomy. 4–5 µm thin tissue slices were cut with a microtome and stained with Hematoxylin–Eosin (H&E). The slides were examined and photographed with a light microscope (DIALUX 20 EB) at 40X.

#### Statistics

Data are expressed mean ± SEM (*n* = 8). One-way analysis of variance (ANOVA) followed by Tukey’s test was applied for calculating the statistical changes among different experimental groups using Graph pad prism 5 software. The level of significance was set at *p* < 0.05.

## Results

### Effect on body weight

The effect of CisPT, and pretreatments with AHE on the rat body weight was presented in Table 1. The inoculation of CisPT did not exterminate any rats in the course of the testing. However, following the CisPT injection, the animals showed significantly (*p *< 0.001) reduced body weight compared to a stable weight improvement in control rats. The pretreatment of AHE in CisPT treated animals significantly increases body weight in a dose-dependent manner, and a 400 mg/kg dose seems to be more effective (*p* < 0.001) in improving the weight loss induced by CisPT. The gain in body weight in rats pretreated with AHE depicts the growth-promoting advantageous health effect of AHE during CisPT treatment in rats.

**Table 1:** Effect of Cisplatin (CisPT) and AHE treatment on body weight of rats

Treatment (mg/kg)	Body weight (g)	
	Initial	Final
**Control**	210.0 ± 0.427	253.3 ± 0.523^++^
**CisPT**	211.3 ± 0.617	222.3 ± 0.551^**^
**AHE alone**	210.3 ± 0.577	256.3 ± 0.721^++^
**AHE (200) + CisPT**	215.7 ± 0.812	233.1 ± 0.561^**,++^
**AHE (400) + CisPT**	212.3 ± 0.682	245.7 ± 0.712^*,++,##^
**Sily + CisPT**	214.0 ± 0.537	244.3 ± 0.553^*,++^

Data expressed as mean ± SEM (*n* = 8). Asterisks ^*^, ^**^ indicated a significant difference in the final body weight of the group Vs. Control group at *p* < 0.05 and *p* < 0.001 respectively, ^++^ indicated significant difference of final body weight of group Vs. CisPT-treated group at *p* < 0.001, ^##^ indicated significant difference of final body weight of AHE (200 mg/kg) + CisPT treated group Vs. AHE (400 mg/kg) + CisPT treated group at *p* < 0.001. Non-significant difference (*p* > 0.05) was recorded between the control and AHE alone treated group in all parameters. (One-way ANOVA followed by Tukey’s multiple comparison tests). Sily-Silymarin

### AHE pretreatment precludes cisplatin-interceded upsurge in acetylcholinesterase activity

**Fig. 1:**
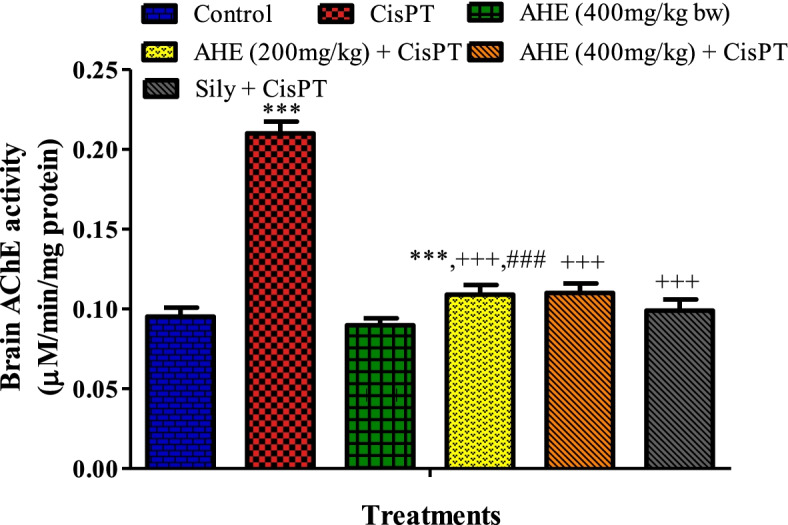
Effect of AHE (200 and 400 mg/kg) on CisPT-induced changes in the activities of (**A**) AChE (μM/min/mg protein) in the brain of rats. Data are shown as the mean ± standard error of mean (*n* = 8). ^*, **, ***^ indicate significance from the control group at *p* < 0.05, p < 0.01 and *p* < 0.0001 probability level, ^+, ++, +++^ indicate significance from the CisPT group at *p* < 0.05, p < 0.01 and *p* < 0.0001 while ^###^ indicate significance of AHE 200 mg/kg group vs AHE 400 mg/kg group at *p* < 0.0001 probability level **(**One-way ANOVA followed by Tukey’s multiple comparison tests). Abbreviations**:** AChE, acetylcholinesterase; CisPT, cisplatin

Figure 1 showed the effect of AHE on AChE activity in the brain of rats treated with CisPT. CisPT inoculation caused a significant increase in AChE activity in the brain of the treated rats whereas pretreatment with AHE (400 mg/kg b.w.) significantly prevented the adverse effect of CisPT as shown by the decrease in AChE action in brain tissue. The effect of AHE is equivalent to the standard drug silymarin.

### Inflammatory biomarkers

**Fig. 2:**
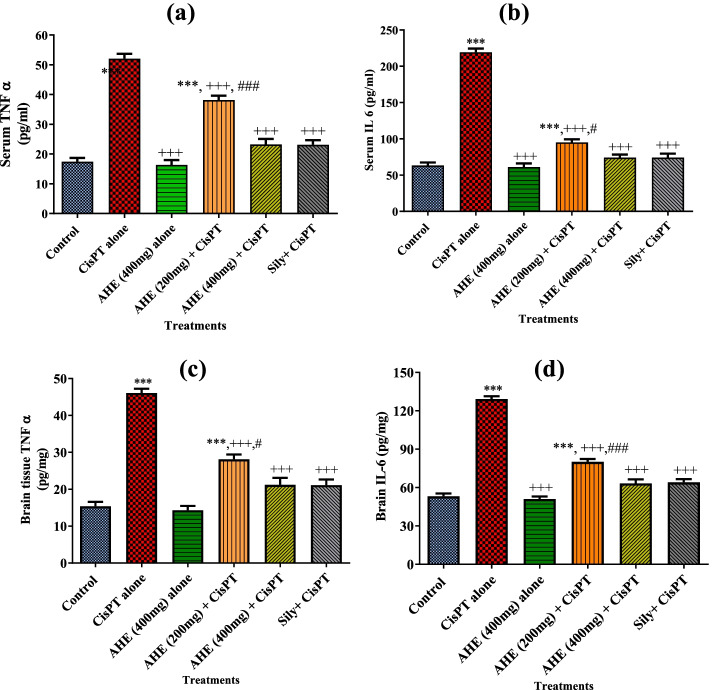
Effect of various treatments on serum and brain anti-inflammatory biomarkers. Upper panel (**a**) and (**b**) serum TNF-α ad IL-6 levels, lower panel (**c**) and (**d**) brain TNFα ad IL-6 levels

Effects of various treatments on serum and brain tissue TNF-α and IL-6 levels measured are shown in Fig. 2. CisPT shot significantly elevated serum and brain tissue inflammatory biomarkers as compared to the untreated group. However, AHE pretreatment significantly ameliorated the activity of TNF-α and reverted pro-inflammatory cytokine IL-6 levels to values near that of control. AHE administration showed substantial preventive potential in a dose-dependent fashion.

### Pretreatment with AHE improved antioxidant defense mechanisms in the brain

Tables 2 and 3 illustrate the effect of AHE pretreatment on the antioxidant defense system in rat brains. CisPT inoculation induced a remarkable (p < 0.0001) reduction in renal POD, SOD, CAT, and QR activity in contrast to untreated rats. However, pretreatment of rats with AHE initiated a substantial escalation of POD, SOD, CAT, and QR levels in comparison to CisPT alone treated group.

**Table 2:** Effect of Cisplatin (CisPT) and different treatments of AHE on Brain POD, SOD, CAT, and QR levels

Treatment (mg/kg)	POD (U/min)	SOD (U/mg protein)	CAT (U/min)	QR (nM/min/mg protein)
Control	11.85 ± 0.130^+++^	1.812 ± 0.060^+++^	35.50 ± 0.289^+++^	93.49 ± 0.526^+++^
CisPT	5.170 ± 0.383^**^	0.541 ± 0.050^***^	18.43 ± 0.251^***^	63.55 ± 1.275^***^
AHE alone	11.30 ± 0.185^+++^	1.831 ± 0.071^+++^	35.93 ± 0.479^+++^	93.84 ± 0.140^+++^
AHE (200) + CisPT	8.010 ± 0.151^**,+++^	1.165 ± 0.098^**,+++^	26.96 ± 0.248^***,+++^	73.32 ± 0.667^***,+++^
AHE (400) + CisPT	10.14 ± 0.203^+++,##^	1.697 ± 0.083^+++,##^	30.08 ± 0.107^*,+++,##^	83.22 ± 0.491^**,+++,##^
Sily + CisPT	10.12 ± 0.196^+++^	1.675 ± 0.066^+++^	30.19 ± 0.335^*,+++^	83.11 ± 0.537 ^**,+++^

Data expressed as mean ± SEM (*n* = 8). Astericks ^*^, ^**^, ^***^ indicted significant difference of final body weight of group Vs. Control group at *p* < 0.05, *p* < 0.001 and *p* < 0.0001 respectively, ^++, +++^ indicated significant difference of final body weight of group Vs. CisPT-treated group at *p* < 0.001 and *p* < 0.0001 respectively, ^##^ indicated significant difference of final body weight of AHE (200 mg/kg) + CisPT treated group Vs. AHE (400 mg/kg) + CisPT treated group at *p* < 0.001. Non-significant difference (*p* > 0.05) was recorded between control and AHE alone treated group in all parameters. (One-way ANOVA followed by Tukey’s multiple comparison tests). Sily-Silymarin

**Table 3:** Effect of CisPT and different treatments of AHE on brain GSH profile and phase II antioxidants

Treatment (mg/kg)	GSH (µM/g tissue)	GR (nM/min/mg protein)	GST (nM/min/mg protein)	γ-GT (nM/min/mg Protein)	GPx (nM/min/mg Protein)
Control	18.01 ± 0.529^+++^	155.4 ± 0.625^+++^	139.3 ± 0.375^+++^	397.4 ± 1.446^+++^	130.7 ± 1.300^+++^
CisPT	9.122 ± 0.323^***^	94.22 ± 1.480^***^	92.60 ± 0.756^***^	109.9 ± 1.166^***^	74.85 ± 0.964^***^
AHE alone	18.28 ± 0.673^+++^	154.9 ± 0.409^+++^	139.5 ± 0.452^+++^	398.1 ± 1.379^+++^	130.9 ± 1.101^+++^
AHE (200) + CisPT	12.83 ± 0.145^***,+++^	122.2 ± 0.779^***,+++^	111.1 ± 0.288^***,+++^	218.2 ± 0.672^***,+++^	96.00 ± 1.134^***,+++^
AHE (400) + CisPT	17.13 ± 0.302^+++,##^	142.1 ± 1.146^**,+++,##^	133.1 ± 0.853^*,+++,###^	380.9 ± 0.610^+++,###^	126.3 ± 1.052^+++,###^
Sily + CisPT	17.00 ± 0.145^+++^	141.0 ± 1.201^**,+++^	132.8 ± 1.049 ^*,+++^	381.5 ± 0.691^+++^	125.6 ± 0.985^+++^

Data expressed as mean ± SEM (*n* = 8). Astericks ^*^, ^**^, ^***^ indicted significant difference of final body weight of group Vs. Control group at *p* < 0.05, *p* < 0.001 and *p* < 0.0001 respectively, ^++, +++^ indicated significant difference of final body weight of group Vs. CisPT-treated group at *p* < 0.001 and *p* < 0.0001 respectively, ^##, ###^ indicated significant difference of final body weight of AHE (200 mg/kg) + CisPT treated group Vs. AHE (400 mg/kg) + CisPT treated group at *p* < 0.001 and *p* < 0.0001 respectively. Non-significant difference (*p* > 0.05) was recorded between control and AHE alone treated group in all parameters. (One-way ANOVA followed by Tukey’s multiple comparison tests). Sily-Silymarin

Furthermore, CisPT alone considerably decreases brain tissue GSH, GR, GST, γ-GT, and GPx content. On the other hand, pretreatment with AHE substantially (*p* < 0.001) prevented CisPT-persuaded diminution in the phase II antioxidant enzymes in a dose-dependent manner. GSH, GR, GST, γ-GT, and GPx activity were highly improved in 400 mg/kg AHE and silymarin-administered animals. Depletion of Glutathione act as a major contributing factor involved in brain injury. We noticed that AHE administration at 400 mg/kg dose rescued GSH content near to the normal range. Whereas significant improvement in the activity of GR, GST, γ-GT, and GPx was provided by AHE in comparison to the CisPT group.

### AHE precluded CisPT triggered oxidative stress

MDA, NO, and H_2_O_2_ are the main oxidative trauma indicators. CisPT dosage noticeably reduced protein content in brain tissues while amplifying the activity of oxidative biomarkers (MDA, H_2_O_2_, and NO) in contrast to control. AHE works in dose-dependent mode as 400 mg/kg dose retrieved the brain protein, NO, H_2_O_2_, and MDA content more efficiently than the amounts similar to the control group. The AHE 400 mg/kg dose produced similar effects as the standard drug silymarin. AHE administration in both low and high doses before CisPT-inoculation minimized oxidative stress in contrast to the CisPT alone treated group (Table 4).

**Table 4:** Effect of CisPT and different treatments of AHE on brain tissue protein, oxidative stress markers and lipid peroxidation

Treatment (mg/kg)	Protein (µg/mg Tissue)	H_2_O_2_ (nM/min/mg Tissue)	Nitrite (content µM/ml)	TBAR (nM/min/mg protein)
Control	2.330 ± 0.046^+++^	1.743 ± 0.039^+++^	47.84 ± 1.156^+++^	5.026 ± 0.301^+++^
CisPT	0.801 ± 0.125^***^	4.946 ± 0.051^***^	85.00 ± 1.055^***^	11.92 ± 0.321^***^
AHE alone	2.370 ± 0.019^+++^	1.707 ± 0.013^+++^	45.46 ± 1.302^+++^	5.007 ± 0.183^+++^
AHE (200) + CisPT	1.81 ± 0.045 ^***,++^	3.381 ± 0.036^***,+++^	66.73 ± 1.620^***,+++^	8.643 ± 0.279^***,+++^
AHE (400) + CisPT	2.298 ± 0.076^+++,##^	2.04 ± 0.047^**,+++,###^	50.84 ± 1.051^+++,###^	6.119 ± 0.077^+++,###^
Sily + CisPT	2.285 ± 0.039^+++^	2.109 ± 0.07^**,+++^	49.13 ± 1.075^+++^	6.171 ± 0.191^*,###^

Data expressed as mean ± SEM (*n* = 8). Astericks ^*, **, ***^ indicted significant difference of final body weight of group Vs. Control group at *p* < 0.05, *p* < 0.001 and *p* < 0.0001 respectively, ^++, +++^ indicated significant difference of final body weight of group Vs. CisPT-treated group at *p* < 0.001 and *p* < 0.0001 respectively, ^##, ###^ indicated significant difference of final body weight of AHE (200 mg/kg) + CisPT treated group Vs. AHE (400 mg/kg) + CisPT treated group at *p* < 0.001 and *p* < 0.0001 respectively. Non-significant difference (*p* > 0.05) was recorded between control and AHE alone treated group in all parameters. (One-way ANOVA followed by Tukey’s multiple comparison tests). Sily-Silymarin

### Evaluation of DNA damage

#### DNA ladder test

**Fig. 3:**
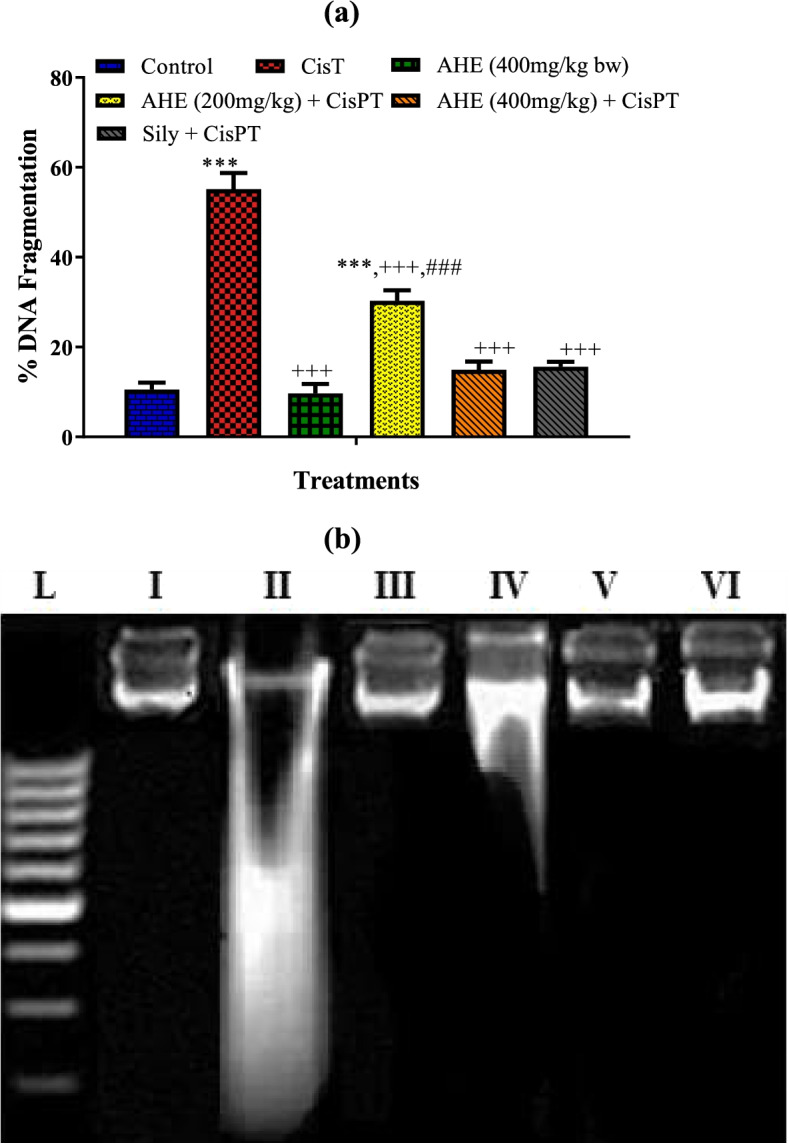
(**a**) DNA fragmentation (%) in different treatment groups, (**b**) Agarose gel showing DNA damage by CisPT and protective effects of Pretreatment of AHE and silymarin in brain tissue. Lanes from left (M) low molecular weight marker, (I) control, (II) CisPT group, (III) AHE (400 mg/kg bw) group, (IV) AHE (200 mg/kg) + CisPT (V) AHE (400 mg/kg) + CisPT (VI) Sily + CisPT group

Gel electrophoresis represented different band patterns in various groups (Fig. 3a). The genomic DNA presented distinct bands with no smearing in the control group. CisPT caused damage to brain genomic DNA that appear as smearing as well as a specific pattern of band fragmentation that was visibly different from the control group. We observed obliteration of bands for the amplified DNA retrieved from CisPt group. AHE 400 mg /kg dose offers more evident repairing of the DNA damage. A comparable effect was recorded with the Silymarin administration. The group treated with AHE alone did not display any signs of DNA injuries.

#### AHE inhibited CisPT-persuaded % fragmentation of DNA

DNA fragmentation (%) pattern explicit clear changes in various groups (Fig. 3 b). CisPT induced significant DNA fragmentation that indicated instability of genomic DNA after CisPT administration. The AHE administration prevented DNA instability by lowering % DNA fragmentation revealing the protective potential at the genetic level. A high dose (400 mg/kg) of AHE predisposes comparable protection to the Silymarin-treated group and the percent DNA fragmentation (%) in both groups was near to the control group.

#### Histopathology

**Fig. 4:**
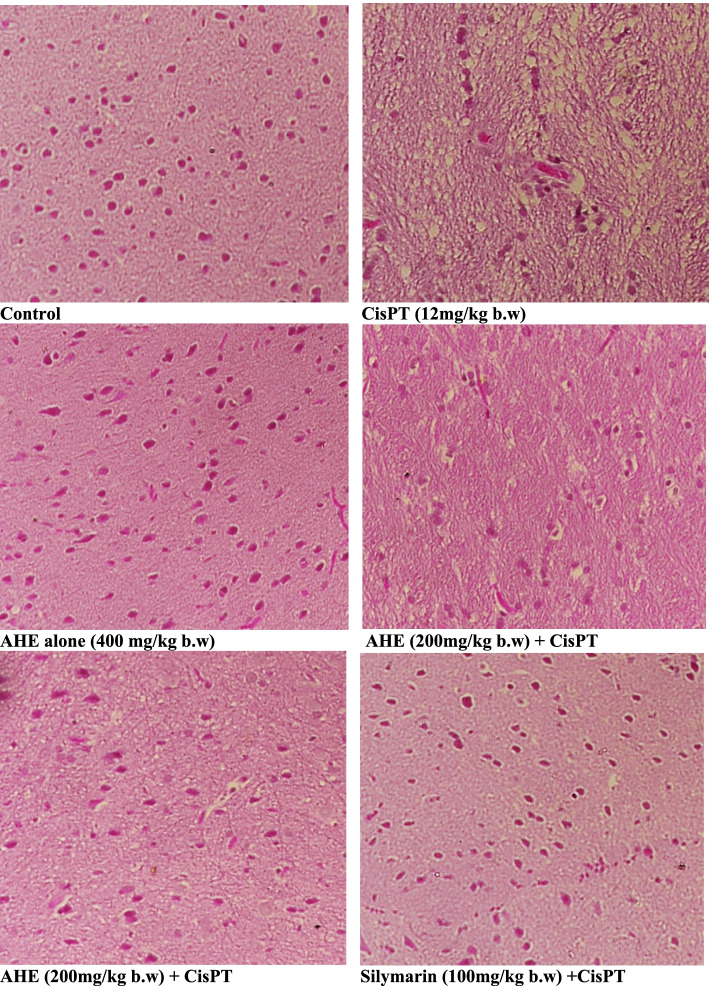
Cerebral cortex sections from the untreated group showing normal histological features of brain morphology with well-shaped neurons. Cisplatin (CisPT, 12 mg/kg) shot induces severe damage in cerebral cortex regions. Sections of *Acacia hydaspica* ethyl acetate extract (AHE) pretreated group at 200 mg/kg for 16 days showed significant protection and rescue of neuron damages with slight signs of the CisPT damage tissues. Sections of AHE (400 mg/kg) pretreated for 16 days showed brain regions with fewer damaged tissues than CisPT group and showed near-to normal brain morphology with well-organized nuclei devoid of vacuolation and irregular features

Figure 4 shows the histopathological changes observed in different experimental groups. The control and AHE alone groups showed characteristic tissue morphology with cellular size and intensely basophilic Purkinje cells. CisPT inoculation induced obvious histological damage in the brain tissue. The Purkinje cells of the CisPT group were shrunken with some exhibiting features of karyolysis and had peri-cellular halos. Many vacuoles of variable sizes were observed in maximum numbers of cells. Treatments with AHE considerably rescued brain tissue from the toxic effects of CisPT in a dose-dependent mode. Few darkly stained nuclei, pericellular halos, and the vacuolated neuropil were observed. The evident protection of morphological features was recorded in the 400 mg /kg AHE treatment group.

## Discussion

Neurotoxicity is a common adverse effect that has been detected in nearly 50% of the patients treated with CisPT, due to this clinical use of CisPT has been limited. Explication of mechanisms involved in CisPT neurotoxicity is essential to discover potential complementary remedies. The exact mechanism of CisPT inducing neurotoxicity is still under investigation however, oxidative trauma, DNA damage and inflammation appear to be the main mechanisms [38]. Antioxidant composites have shown compelling neuroprotective actions both in vitro and in vivo [39]. Previous studies had testified that AHE stimulated antioxidant levels via inhibition of lipid peroxidation and inflammation in rats [3, 40, 41]. In the current investigation two doses of AHE were tested (200 and 400 mg/kg. b.w) and results showed that both doses of AHE, especially the high dose, were potent in lessening the neurotoxic effect of CisPT on the brain. AHE considerably restored the deteriorated brain antioxidant levels, and DNA damages, and suppressed the neuro-inflammation and histological alterations.

Acetylcholinesterase governs cholinergic neurotransmission by hydrolyzing acetylcholine at synapses [37]. Cholinergic deterioration is a principal sign of neurotoxicity. The marked increase in the AChE activity observed in CisPT-treated rats eventually leads to decreased acetylcholine amount in the synaptic cleft and thus impairs the normal neurotransmission which may be linked to the decreased motor function. AHE pretreatment ameliorated AChE activity in a dose-dependent manner. The marked reduction in acetylcholinesterase activity in rats pretreated with AHE suggests the preventive role of AHE in CisPT-mediated neurotoxicity in rats. The AChE inhibiting action of AHE depicts the potential of this plant in the treatment of Alzheimer’s disease as well. Amplified generation of ROS may be one of the reasons that account for disturbed AChE activity which affects the functions of the cholinergic system. The observed protective effect of AHE might be linked to the occurrence of the rich source of flavonoids in AHE, in particular, catechin, which might be liable for its cholinesterase inhibitory and antioxidant activities. Previous research indicated that catechin is brain permeable and possesses neuroprotective actions [42, 43]. Similarly, a study conducted by Crowch et al. demonstrated the acetylcholinesterase inhibitory property of Acacia nilotica in vitro models.

The brain is highly vulnerable to oxidative trauma because of its maximum utilization of oxygen, and increase quantity of non-heme iron which enhances the generation of ROS that outcome in damaging of various subcellular macromolecules. CisPT can disturb redox homeostasis and this fact is similarly convinced in the present study. We noticed an enhanced level of H_2_O_2_, lipid peroxidation and NO levels in the brain of CisPT treated rats. NO is a strong damaging free radical that can dynamically react with lipids, proteins, and DNA, and besides it inactivates GSH and GPx [44]. Our findings are in agreement with previous findings indicating an increase in oxidative stress biomarkers in the brain of CisPT-treated rats [6]. Furthermore, CisPT induced a noticeable decrease in the brain SOD, CAT, and GST expression with a simultaneous reduction in GSH quantity indicating compromised antioxidant defense mechanisms in the brain. After CisPT injection, platinum sulfhydryl group complexes are absorbed by neuronal cells and naturally alleviated by intracellular GSH for several hours. However, under intracellular GSH depletion the complexes endure prompt alteration to reactive metabolites [20]. GSH depletion also results in Lipid peroxidation and overall compromised antioxidant defense system. These observations support the hypothesis that the mechanism of neurotoxicity in CP‑treated animals is related to the depletion of the antioxidant defense system [39, 40]. CisPT-induced changes in MDA, H2O2, and NO levels were markedly prevented by pretreatment with AHE in a dose-dependent fashion. Besides, AHE pretreatment augments brain antioxidant enzyme activities and GSH levels thus indicating the beneficial effect of AHE against CisPT-induced brain oxidative damage. The ameliorative effect of AHE may infer its aptitude to reinstate the normal brain oxidative levels by eradicating free radicals and antioxidant properties. A previous report revealed the potent radical scavenging and metal chelating potential of AHE [45].

Neuro-inflammation is the major mechanism in CisPT-induced brain damage [46]. Among the pro-inflammatory cytokines that trigger the CisPT-mediated pathophysiological developments associated with brain injury; TNF-α and IL-6 are extremely potent as they also stimulate the discharge of other cytokines as well. This was evident in our study by the increase in the levels of TNF-α and IL-6 in both the brain and the serum which agrees with the previous study. In our study, high levels of pro-inflammatory cytokines induced by CisPT showed a worthy response to pretreatment with AHE as evident by the drop in the levels of TNF-α and IL-6 in the brain as well as serum. Therefore, the anti-inflammatory perspective of AHE by deactivating TNF-α and IL-6 and its antioxidant activity by enhancing GSH might be the imperative mechanisms behind the observed protective effect of AHE. Besides other bioactive compounds, AHE is rich in catechins and reports have testified to the anti-inflammatory properties of catechins. EGCG impedes the release of vascular endothelial growth factors IL-6, and IL-8 in human astrocytoma (U373MG cells). Anti-inflammatory activities of various cytokines are suppressed by EGCG by a decline of IL-1β and Aβ-induced COX-2 expression [47]. EGCG impedes LPS-stimulated microglial activation and protects against neuronal injury triggered by inflammation [48]. So the occurrence of catechin in AHE might be responsible for the noticed anti-inflammatory activity in brain tissue.

Histopathological study corresponds to the biochemical findings. We observed severe degenerative alterations in CisPT treated group with severe dystrophic and apoptotic variations in the neurocytes of the cerebral cortex and hippocampus. Brain tissue histology revealed deterioration in the cerebellum of rats as illustrated by shrinkage and reduction of the size of the Purkinje neurons [13, 24]. CisPT supposedly interacts with DNA via the creation of covalent adducts among certain DNA and the platinum compound resulting in cellular toxicity [42] and consequently neuronal death. CisPT exposure leads to relative weakness and death of Purkinje neurons in the treated rats. The vacuoles in the neighboring neuropil might be attributed to the shrinkage of cells and removal of their secondary processes leaving pericellular spaces. Besides, CisPT mediated deteriorating changes could be an upshot of decrease in the amount of brain antioxidant enzymes and neurons due to widespread neurodegeneration. On the other hand, pretreatment of rats with AHE prevented CisPT-induced changes in the brain tissue. AHE administration (200 and 400 mg/kg) showed marked reduction in the neurodegeneration with observable improvement in neuronal morphology and regeneration of neurons. AHE rescued CisPT-induce alterations due to its antioxidant potential and phytoconstituents. Previous study reported that epicatechin exhibited increased levels of endogenous antioxidants, protective against lipid peroxidation, prevented neuronal death and neuronal necrosis, and accuracy of brain tissue morphology [49].

## Conclusion

Biochemical and histological data indicated that CisPT-induced brain damage involved inconsistency in the ratio of brain tissue oxidant and antioxidant, and AHE showed protection against CisPT-induced Brain impairments via reversing the altered levels of CisPT-induced enhanced activities of AChE, altered oxidative/antioxidative status, DNA damage and enhanced production of pro-inflammatory cytokines, suggesting a potential role for AHE in CisPT-induced brain toxicity.

### Limitations of the study

Large-scale prospective studies are obligatory to approve the biochemical results obtained in our study using molecular tools and more immune-histochemical techniques. For this evaluation of the gene expression of GSH, AChE, TNF-α, IL-1β, and IL-6 using PCR and their protein expression using western blotting is suggested. Besides we were unable to compare the current findings with others because of a lack of investigations on the protective effects of Acacia hydaspica against drug-induced brain damage. Demonstration of the possible behavioral benefits of reducing oxidative stress with AHE is recommended in the future.

## Data Availability

The datasets used and/or analyzed during the current study are available from the corresponding author on reasonable request.

## Abbreviations

CisPT: Cisplatin
AHE: *Acacia **hydaspica* Ethyl acetate extract
AChE: Acetylcholinesterase (AChE) activity
IL-1 β: Interleukin-1-beta
IL-6: Interleukin- 6
TTE: Tris-TAPS-EDTA
TCA: Trichloroacetic acid
EDTA: Ethylenediaminetetraacetic acid
PCR: Polymerase Chain Reaction
TNF-α: Tumor necrosis factor
GSH: Glutathione
POD: Peroxidase
CAT: Catalase
ROS: Reactive oxygen species
MDA: Malondialdehyde
H_2_O_2_: Hydrogen peroxide
NO: Nitric oxide
